# Transcriptomic sequencing reveals dynamic shifts in nutrient utilization and energy metabolism during embryonic and larval development of the purple sea urchin *Heliocidaris crassispina*

**DOI:** 10.1186/s12864-026-13190-z

**Published:** 2026-07-30

**Authors:** Zepeng Zhang, Fanjiang Ou, Jianfeng Xu, Zhou Qin, Wenjie Pan, Yi Zhang, Youkai Tu, Jiasheng Huang, Yang Liu, Aifen Yan, Haipeng Qin, Chunhua Ren, Chaoqun Hu, Hongyan Sun, Xiao Jiang, Ting Chen

**Affiliations:** 1 Guangdong Laboratory for Lingnan Modern Agriculture, College of Marine Sciences, South China AgricultuCral University, Guangzhou, 510642 People’s Republic of China; 2 State Key Laboratory of Breeding Biotechnology and Sustainable Aquaculture, Laboratory of Tropical Marine Bio-Resources and Ecology, Guangdong Provincial Key Laboratory of Applied Marine Biology, South China Sea Institute of Oceanology, Chinese Academy of Sciences, Guangzhou, 510301 People’s Republic of China; 3 School of Medicine, Foshan University, Foshan, 528225 China; 4 Agro-Tech Extension Center of Guangdong Province, Huizhou, 516081 China

**Keywords:** Sea urchin, Embryonic and larval development, Transcriptome, Gene expression

## Abstract

**Background:**

*Heliocidaris crassispina* is an economically important sea urchin in the Northwest Pacific, yet large-scale aquaculture is constrained by high mortality during early development. This limitation is largely attributable to the poor understanding of gene expression patterns across these critical stages. Therefore, a stage-resolved developmental transcriptomic profile from fertilization to the settled juvenile is needed to clarify the molecular basis of early developmental transitions and to support improved hatchery management and stage-appropriate feeding.

**Results:**

We described a complete developmental profile of *H. crassispina* from fertilization to the settled juvenile and established transcriptomes for 14 consecutive developmental stages. Beginning with the two-arm pluteus stage, *H. crassispina* undergoes rapid growth, paralleling a marked rise in mortality that continues through metamorphosis until the juvenile settles. Based on global gene expression patterns, development was divided into four periods. Period-I spanned the fertilized egg to 32-cell stages, Period-II covered two late-cleavage stages (LC1–LC2), Period-III extended from blastula to two-arm pluteus, and Period-IV extended from the four-arm pluteus to the settled juvenile stages. GO and KEGG analyses indicated a stepwise transition in gene expression programs across developmental periods, consistent with the morphological and physiological changes observed. Weighted gene co-expression network analysis (WGCNA) identified ten modules whose expression profiles correlated significantly with specific developmental periods, indicating development is regulated by a coordinated, module-level transcriptional program. Analysis of digestive enzyme and energy metabolism genes showed that early embryos rely on endogenous reserves, whereas feeding larvae progressively establish exogenous digestive capacity, accompanied by stage-dependent fluctuations and a late-stage rise in glycolysis and TCA cycle gene expression, suggesting a metabolic transition associated with exogenous feeding and settlement.

**Conclusion:**

This study established transcriptomes for 14 consecutive stages of *H. crassispina* from fertilization to the settled juvenile and, together with growth and survival, resolved four transcriptomic periods. Functional enrichment and WGCNA indicated coordinated, period-specific programs that track major morphological and physiological transitions. Digestive enzyme and energy metabolism genes showed a clear shift from reliance on endogenous reserves in embryos to the progressive establishment of exogenous digestive capacity in feeding larvae, accompanied by late-stage upregulation of glycolysis and TCA cycle genes around settlement. These insights support stage-specific feeding and hatchery management to reduce mortality.

**Supplementary Information:**

The online version contains supplementary material available at https://doi.org/10.1186/s12864-026-13190-z.

## Introduction

Sea urchins, as representative commercial echinoderms, are highly valued in the market because their gonads are nutrient-rich and possess a distinctive flavor, making them an important marine fishery resource [1, 2]. Sea urchins also play critical ecological roles in marine ecosystems by grazing on macroalgae and consuming detritus, thereby contributing to the health of coral reefs and kelp forests [3]. In recent years, sustained growth in market demand has led to overharvesting and a severe decline in wild sea urchin stocks across multiple regions, underscoring the need for aquaculture to stabilize supply and reduce fishing pressure on natural populations [4, 5].

**Fig. 1:**
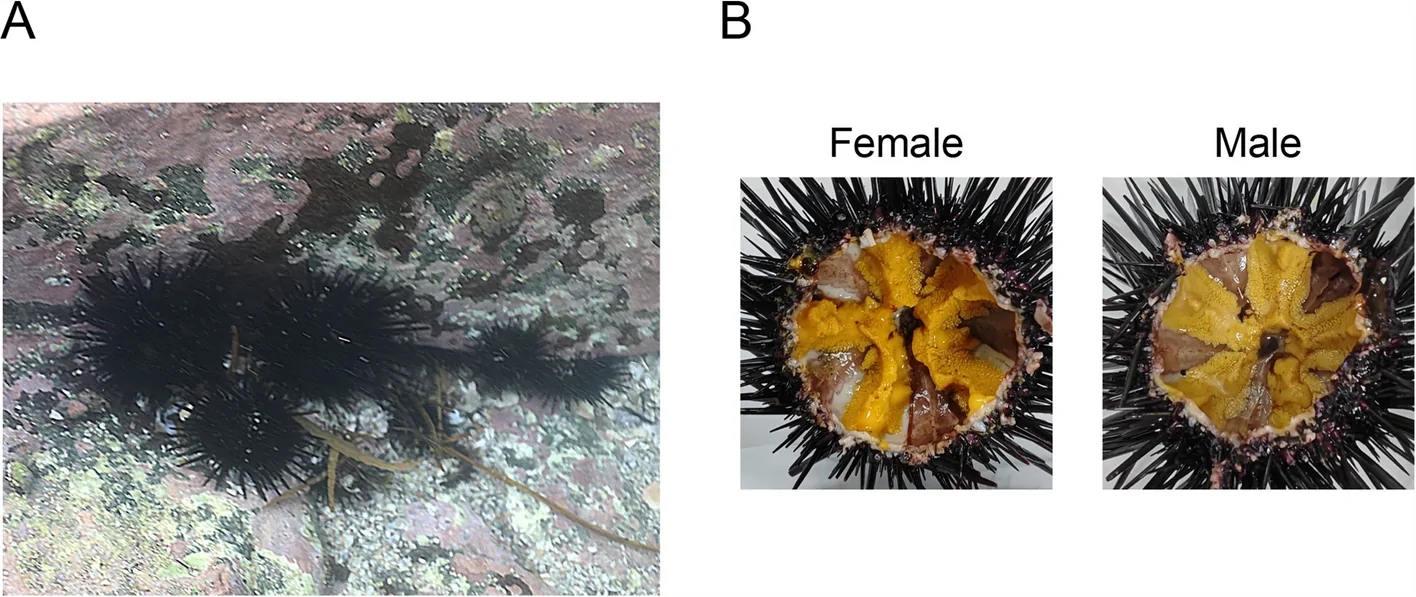
Representative photographs of the purple sea urchin *H. crassispina*. **A** Adult individuals in a rocky habitat. **B** Dissected gonadal tissue from female and male specimens

The purple sea urchin (*Heliocidaris crassispina*) is primarily distributed in the tropical and subtropical waters of the Northwest Pacific, including the coastal regions of China, Japan, and other neighboring countries [3, 6, 7] (Fig. 1A). With high levels of polyunsaturated fatty acids, both the ovary and testis of *H. crassispina* are regarded as premium seafood (Fig. 1B), resulting in strong market demand [8]. Therefore, increasing attention has been directed to the development of aquaculture for *H. crassispina* [9]. Although artificial spawning of *H. crassispina* has been achieved, large-scale production remains constrained by low survival during early development from fertilization to settlement and juvenile formation [10]. In this study, early development refers to the period from fertilization to the settled juvenile stage, including embryonic development, planktonic larval development, metamorphosis, and settlement. To address these challenges, it is essential to understand the developmental characteristics of *H. crassispina* embryos and larvae, along with their underlying gene-expression regulatory mechanisms.

In sea urchin larval production, substantial losses frequently occur during the embryonic and early larval stages [4]. In sea urchins and other echinoderms, the proportion of fertilized eggs that successfully complete cell division, develop into planktonic larvae, undergo metamorphosis, and ultimately settle as juveniles is typically very low, making early development a major bottleneck for population recruitment in these species [11, 12].

The survival and settlement rates of sea urchin larvae in the natural environment are markedly lower than those observed under laboratory conditions, which further suggests that the planktonic larval stage constitutes a critical limiting phase for individual development [13]. Under controlled conditions, changes in salinity, nutrient supply, and seawater component can strongly affect embryonic development and larval growth, and may even lead to developmental arrest and mortality [14, 15]. Both food availability and pH fluctuations can critically influence larval development, inducing phenotypic and molecular responses, or altering developmental patterns [15, 16]. Moreover, comparative analysis of different hatchery conditions highlighted the high sensitivity of early embryos to variations in ionic composition and water chemistry [17]. Taken together, it can be speculated that the high vulnerability of early development in *H. crassispina* may be linked to the transition during depletion of maternal reserves, before the new regulatory networks related to nutrient acquisition and energy metabolism are fully established. Therefore, identifying the key developmental transition phases in *H. crassispina* early development and elucidating the transcriptional regulation of associated genes will provide valuable guidance for optimized larval rearing.

In this study, we documented the complete developmental progression of *H. crassispina* from fertilization to the settled juvenile stage and profiled the transcriptomes across 14 consecutive developmental stages. By integrating principal component analysis (PCA), differential expression gene (DEG) analysis, and weighted gene co-expression network analysis (WGCNA), we delineated stage-specific gene expression and examined their correlation with the onset of nutrient acquisition and metabolic regulation. We further analyzed the expression dynamics of digestive enzyme-related genes and key energy-metabolism pathways throughout development, to elucidate the molecular mechanisms governing the transition from maternal provisioning to exogenous feeding. Together, our findings identify key transcriptional transitions during early development in *H. crassispina*, and provide a molecular framework for optimizing larval rearing and stage-specific culturing of this species.

## Materials and methods

### Parental collection and spawning induction

Adult purple sea urchins (*Heliocidaris crassispina*) used in this study were collected from Daya Bay (22°40.1’ N, 114°39.2’ E), Shenzhen City, Guangdong Province, China. A total of 100 individuals were obtained by diving. After collection, the animals were immediately transported to the Guangdong Marine Fisheries Development Center (Huizhou City, Guangdong Province, China) for acclimation and were fed with macroalga. During acclimation, seawater temperature was maintained at 29.1 ± 1.0 °C and salinity was approximately 30.3 ppt. After 7 days, spawning was conducted with *H. crassispina* with a diameter of 4.6 ± 0.3 cm and a wet weight of 74.3 ± 1.0 g.

Following a standard sea urchin spawning protocol, female and male adults were induced to spawn via injection of 0.5 mol L^−1^ KCl through the peristomial membrane [17, 18]. After gamete release, 0.1 mL of sperm suspension was assessed microscopically for motility and concentration. For each biological replicate, gametes from three females and three males were used for fertilization. Sperm and egg suspensions were mixed at a 1:10 (v/v) ratio and gently stirred with a glass rod. After 10 min, fertilization envelope formation was confirmed microscopically. Fertilized eggs were then rinsed with clean seawater to remove excess sperm and evenly distributed into a 1200-L culture tank at an initial density of approximately 1 individual mL^−1^. Two air stones were placed at the bottom to provide continuous aeration. During culture, water temperature was maintained at 27.1–29.3 °C, pH at approximately 8.3, and salinity at approximately 30 ppt.

### Larval culture and sampling

After developing into the planktonic larval stage, larvae were maintained on daily feeds of freshly cultured *Chaetoceros muelleri*. Natural seawater was sand-filtered and sterilized prior to use. Algal suspension was supplied initially at 3 L per day (approximately 3 × 10^6^ cells mL^−1^). Subsequently, larvae were fed twice daily (morning and evening). Before each feeding, larval growth and feeding activity were observed microscopically, and the ration was gradually increased with developmental progress. During the planktonic phase, one-third of the culture water was replaced every two days, during which bottom sediments were removed by siphoning while continuous aeration was maintained.

When larvae reached the eight-arm pluteus, settlement substrates were introduced into the culture tank. These consisted of corrugated plates whose surfaces had been preconditioned with a diatom biofilm of *Navicula* sp. The water-exchange regimen was then adjusted to replace one-half of the culture volume daily. During water exchange, debris and sediments at the bottom were removed by siphoning, and aeration was maintained. After approximately 23 days, larvae completed metamorphosis and settled onto the corrugated plates, thereby developing into juveniles.

The abbreviations used in this study refer to transcriptomic sampling stages defined by observable morphology. Embryos, larvae, and juveniles were sampled at 14 developmental stages, including fertilized egg (FE), 2-cell (2C), 4-cell (4C), 16-cell (16C), 32-cell (32C), late cleavage I (LC1), late cleavage II (LC2), blastula (BL), gastrula (GS), two-arm pluteus (DL), four-arm pluteus (TL), six-arm pluteus (SL), eight-arm pluteus (OL), and settled juvenile (SU). The late cleavage I stage (LC1) was defined as a sequential late-cleavage sampling point after the 32-cell stage and before blastula formation, whereas the late cleavage II stage (LC2) was defined as a later sequential late-cleavage sampling point between LC1 and blastula. At each stage, samples were collected using a 300-mesh sieve, and approximately 1.5 mL of embryo, larval, or juvenile suspension was transferred into cryovials with RNase-free consumables. Three biological replicates were prepared for each stage (*n =* 3), each consisting of pooled individuals. The number of individuals pooled per replicate was adjusted across stages to ensure sufficient RNA yield. Approximately 2,000 to 5,000 individuals were used for embryonic stages, 500 to 800 for planktonic larval stages, and 20 to 30 for the settled juvenile stage. All samples were immediately snap frozen in liquid nitrogen and stored at − 80 °C until use.

### Measurement of survival rate and body-length

Survival was monitored throughout development and calculated as the percentage of individuals alive at a given stage relative to the initial number at fertilization. At each sampling point, the culture water was gently mixed to evenly suspend embryos or larvae while minimizing mechanical disturbance. For each culture tank, at least three independent 50-mL subsamples were collected from different positions of the tank. Surviving individuals in each subsample were counted under a light microscope (SOPTOP, EX20), and the mean density of surviving individuals was calculated. The total number of surviving individuals in each tank was estimated by multiplying the mean density by the total culture volume. Survival rate was then calculated as the estimated number of surviving individuals at each developmental stage divided by the initial number of fertilized eggs, multiplied by 100%.

Images of randomly selected healthy individuals were captured under a light microscope. Body length, defined as the maximum linear distance along the anterior–posterior axis, was measured from these images using ImageJ (version 1.54). For each developmental stage, measurements were taken on five biological replicates (*n =* 5), with each replicate representing a single individual. To minimize error, all measurements were performed by the same operator using consistent criteria.

### Data processing

Body length is reported as mean ± SD with *n =* 5. Survival is expressed as a percentage and was calculated from volumetric counts of surviving individuals at each stage based on at least three independent 50-mL subsamples. Data processing and visualization were performed in R using dplyr and ggplot2.

### RNA extraction, library construction, and transcriptome sequencing

Total RNA was extracted using TRIzol (Invitrogen, Thermo Fisher Scientific) according to the manufacturer’s instructions. RNA integrity and purity were assessed by agarose gel electrophoresis and spectrophotometry. mRNA was enriched with Dynabeads mRNA DIRECT or Dynabeads Oligo(dT)_25_ beads (Invitrogen, Thermo Fisher Scientific). Libraries for samples from fertilized egg to eight-arm pluteus were constructed by Novogene using a standard mRNA-seq workflow, which included fragmentation, random-primed cDNA synthesis, AMPure XP bead-based purification and size selection, end repair, A-tailing, adapter ligation, and PCR amplification. These libraries were sequenced on an Illumina NovaSeq X Plus platform (PE150; 2 × 150 bp). Base calling and conversion of BCL files to FASTQ format were performed with bcl2fastq. Juvenile (SU) libraries were sequenced by BGI on a DNBSEQ-T7 platform (PE150; 2 × 150 bp). All datasets were processed through the same bioinformatics pipeline.

### Transcriptome assembly and functional annotation

Raw sequencing data were first quality-controlled using fastp to remove adapter sequences and low-quality reads and then filtered with SOAPnuke v1.6.4 to obtain clean reads. On average, each sample yielded 7.8 Gb of clean data and 51.7 million clean reads. Paired-end clean reads were aligned to the *H. crassispina* reference genome (GCA_044090015.1) [19] using HISAT2 v2.2.1. Transcript assembly was performed with StringTie v2.0, and raw read counts for each gene were generated.

Protein sequences for the *H. crassispina* reference genome (GCA_044090015.1) were obtained from NCBI [19]. Functional annotation was performed through local BLAST (v2.15.0) searches against the Swiss-Prot database (UniProt) and subsequent domain-based annotation using InterProScan (v5.54). Based on these results, genes were mapped to Gene Ontology (GO) terms and Kyoto Encyclopedia of Genes and Genomes (KEGG) pathways for functional classification and enrichment analysis.

### Differentially expressed gene (DEG) analysis

Raw gene-level read counts were used for differential expression analysis, whereas CPM (counts per million) values were calculated in parallel for low-expression filtering and expression-pattern visualization. To reduce background noise, genes with CPM > 1 in at least two samples were retained using the edgeR package. The CPM-based filtered expression matrix was used for hierarchical clustering (pheatmap) and principal component analysis (PCA) to evaluate overall expression similarity among samples. Based on the clustering and PCA results, the 14 developmental stages were further grouped into four transcriptionally distinct developmental periods (Periods I–IV) for downstream comparisons.

Differential expression analysis was conducted on raw counts using DESeq2 (v 1.48.1) with pairwise comparisons between adjacent developmental periods (Period-I vs Period-II, Period-II vs Period-III, and Period-III vs Period-IV). *P*-values were adjusted for multiple testing using the Benjamini–Hochberg method, and genes with |log2 fold change|> 1.5 and padj ≤ 0.01 were defined as differentially expressed genes (DEGs). GO and KEGG enrichment analyses were performed for DEGs, and enrichment results were visualized using ggplot2.

### WGCNA analysis

To identify gene modules with coordinated expression during embryonic and larval development of *H. crassispina*, weighted gene co-expression network analysis (WGCNA) was performed in R using the filtered expression matrix as described above. Pearson correlation coefficients were calculated across all 42 samples, and the soft-thresholding power was selected based on the scale-free topology fit and mean connectivity. A value of β = 10 was chosen for network construction. The correlation matrix was then transformed into an adjacency matrix, followed by construction of a topological overlap matrix (TOM). Average-linkage hierarchical clustering was conducted based on TOM dissimilarity, and gene modules were identified using the dynamic tree cut algorithm. Module eigengenes (MEs) were subsequently calculated, and Pearson correlation analysis between MEs and developmental stages was used to assess module–stage associations. Modules were considered significantly associated with developmental stages at |cor|≥ 0.3 and *P <* 0.05.

### Identification of genes for digestive enzyme, and key enzymes in glycolysis and the TCA cycle

Digestive enzyme genes were identified based on the Swiss-Prot functional annotations obtained as described above. Genes whose annotations explicitly contained digestive enzyme names or corresponding Enzyme Commission (EC) numbers were classified as digestive enzyme genes. Specifically, lipases included monoacylglycerol lipase (MAG), diacylglycerol lipase (DAG), gastric lipase (GPL), and pancreatic lipase (PPL); carbohydrate-hydrolyzing enzymes included α-amylase (AMY), maltase (MAL), maltase-glucoamylase (MGA), lactase (LAC), sucrase-isomaltase (SIS), and trehalase (TRE); proteases included trypsin (TRY) and chymotrypsin (CHY). Based on these criteria, a set of digestive enzyme gene was defined, and their expression were summarized across development to characterize related developmental dynamics. Heatmaps were generated using TBtools-II v2.376.

Reaction steps and key enzyme for glycolysis and the tricarboxylic acid (TCA) cycle were retrieved from the KEGG. Based on the Swiss-Prot functional annotations described above, key genes involved in each reaction step of glycolysis and the TCA cycle were identified, and their expression levels were then summarized across embryonic and larval developmental stages, enabling characterization of the dynamic regulation of energy-metabolism pathways. Expression patterns were visualized using the ggplot2 package in R.

## Results

### Developmental progression and growth dynamics of H. crassispina

**Fig. 2:**
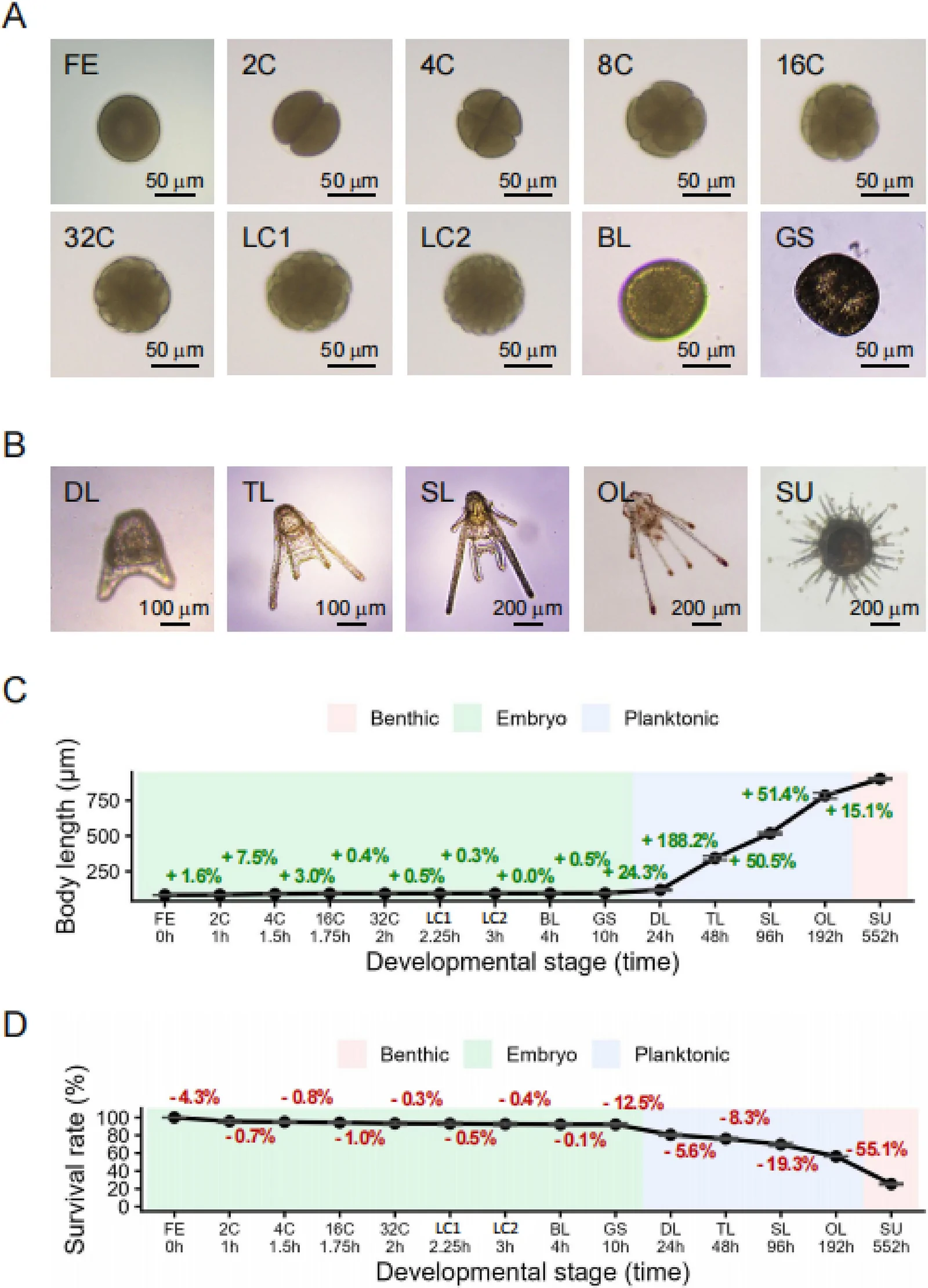
Morphological and growth and developmental characteristics of H. crassispina embryos and larvae.** A** Embryonic stages: fertilized egg (FE), 2-cell (2C), 4-cell (4C), 8-cell (8C), 16-cell (16C), 32-cell (32C), late cleavage I (LC1), late cleavage II stage (LC2), blastula (BL), and gastrula (GS). Scale bar: 50 μm. **B** Planktonic larval and juvenile stages: 2-arm pluteus (DL), 4-arm pluteus (TL), 6-arm pluteus (SL), 8-arm pluteus (OL), and juvenile sea urchin (SU). Scale bars: 100 μm (DL, TL) and 200 μm (SL, OL, SU). Note: The 8 C stage was included for morphological illustration only and was not sampled for transcriptomic sequencing. **C** Body length (µm) across developmental stages. Points represent the mean length at each stage (*n =* 5), and grey error bars indicate SD. Lines connect means of adjacent stages to illustrate the overall growth trend, with growth rates between adjacent stages indicated. **D** Survival rate (%) across developmental stages. Points represent the mean survival rate at each stage, and grey error bars indicate SD based on three replicate measurements. Lines connect means of adjacent stages to illustrate the overall survival trend, with mortality rates between adjacent stages indicated

The early development of *H. crassispina* includes embryonic, planktonic larval, and settlement stages, with the major morphological features and corresponding timing presented in Fig. 2 and Supplementary Table S1 [20].

The first cleavage of the fertilized egg (FE) was completed at approximately 1 h post-fertilization, resulting in two equal-sized blastomeres (2-cell, 2 C). By 1.5, 1.75, 2, and 2.25 h after fertilization, embryos reached the 4-cell (4C), 16-cell (16C), 32-cell (32C), and late cleavage I (LC1) stages, respectively. At approximately 3 h, embryos developed into the late cleavage II stage (LC2), representing a later sequential cleavage-stage sampling point before blastula formation. The blastula stage (BL) and gastrula stage (GS) were reached at 4 and 10 h, respectively, with motile cilia on the outer surface becoming clearly visible and autonomous rotational movement could be observed (Fig. 2A). At 14 h, embryos entered the planktonic larval stage as two-arm pluteus (DL). This transition was marked by clear larval differentiation, the gradual formation of the digestive tract, and a shift from endogenous reserves to exogenous feeding on planktonic microalgae. By 26 h, larvae developed into the four-arm pluteus (TL), characterized by an elongated trunk and four arms. After 4 days, they reached the six-arm pluteus stage (SL), with further increases in body size and feeding capacity. The eight-arm pluteus stage (OL) was attained by 8 days, during which the vestibular complex enlarged markedly. At 23 days, larvae began to settle and attach to substrates, entering the juvenile stage (SU). Juveniles commenced grazing on benthic diatoms, exhibited an adult-like body form, and gradually developed the characteristic black-purple pigmentation during subsequent growth (Fig. 2B).

Body length exhibited distinct stage-specific growth patterns during development (Fig. 2C). During early embryogenesis from the fertilized egg to gastrula stages, embryonic size remained relatively stable, with the maximum diameter ranging approximately from 80 to 100 μm. A marked increase was observed at the larval stage, with the growth rate accelerating notably after the two-arm plutei stage. Upon reaching the eight-arm pluteus stage, larvae attained their maximum body length within the planktonic phase, before their transition into juveniles and benthic settlement (Fig. 2C).

Survival showed an overall declining trend throughout development. During the embryonic stages, the survival rate remained relatively high. After entering the planktonic larval stage, the survival rate began to decrease, with the most serious mortality occurring during the transition from two-arm pluteus to juvenile stages (Fig. 2D). When examined as time series, body length remained stable during embryogenesis but increased in the larval period, whereas survival rate remained stable initially and then declined during larval development. The most pronounced coordinated changes in both traits occurred from after two-arm pluteus to juvenile (Fig. 2C, D).

### Transcriptome sequencing and global expression patterns among samples

A total of 42 libraries were constructed across 14 developmental stages from fertilized egg to juvenile, with three biological replicates. Sequencing generated an average of 7.8 Gb of clean data and 51.7 million clean reads per library, and Q30 values were ≥ 94.57% for all libraries, supporting downstream gene expression analyses.

**Fig. 3:**
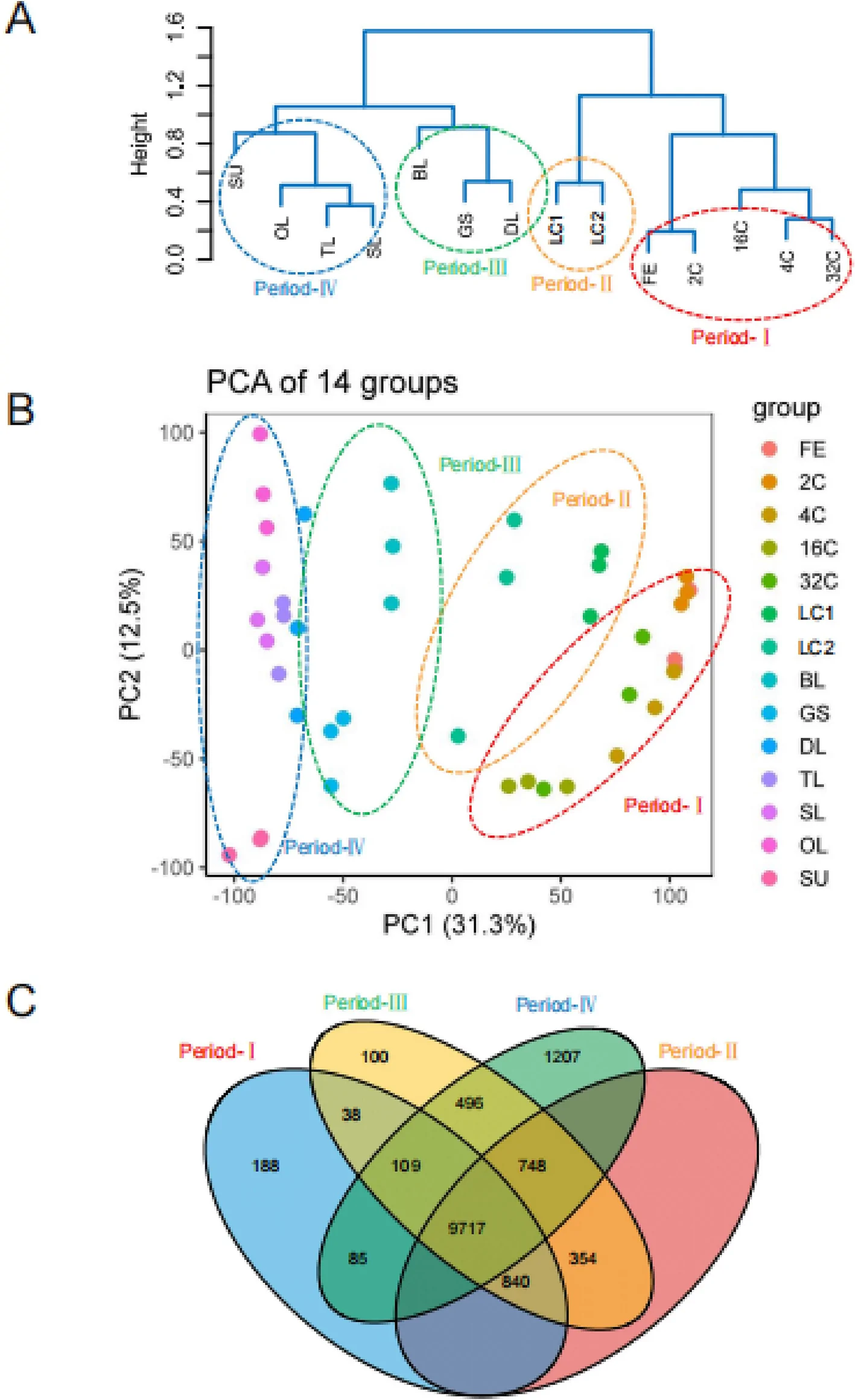
Global gene expression profiles during embryonic and larval development in *H. crassispina*. **A** Hierarchical clustering dendrogram of samples based on a CPM-normalized expression matrix. Four major developmental periods (I–IV) were defined: Period-I (FE to 32 C), Period-II (LC1 to LC2), Period-III (BL to DL, via GS), and Period-IV (TL to SU). **B** Principal component analysis (PCA) of samples from 14 developmental stages based on the CPM-normalized expression matrix. **C** Venn diagram showing the sets of expressed genes in each period. A gene is considered expressed in a given period if its CPM ≥ 1 in at least two-thirds of the biological replicates for that period

To evaluate global expression differences among developmental stages, gene expression clustering (Fig. 3A) and PCA (Fig. 3B) were performed using the CPM-normalized expression matrix. The gene expression clustering tree divided the 14 developmental stages into four distinct branches, namely, fertilized egg to 32-cell Period-I (FE to 32 C), late cleavage I and late cleavage II as Period-II (LC1–LC2), blastula to two-arm pluteus as Period-III (BL to DL), and four-arm pluteus to settled juvenile stages (TL–SU) as Period-IV (Fig. 3A). PCA revealed a clear developmental trajectory along the first two principal components. PC1 (31.3% variance) captured the major progression from fertilized egg to juvenile, with fertilized egg to late cleavage I clustered tightly at one end, late cleavage II and blastula occupied intermediate positions, and gastrula to four-arm pluteus distributed progressively along the axis, and six-arm pluteus stage to juvenile stage formed a distinct cluster at the opposite end. PC2 (12.5% variance) further separated SU from other stages, with biological replicates clustered consistently within each stage (Fig. 3B). This period classification based on gene expression patterns during development can serve as a framework for subsequent DEG and functional enrichment analyses.

Based on this classification, gene expression overlap between periods was compared and visualized using a Venn diagram (Fig. 3C). A total of 9,717 genes were commonly expressed across all four periods. The numbers of period-specific genes were 188 in Period-I, 0 in Period-II, 100 in Period-III, and 1,207 in Period-IV, suggesting increased transcriptional specificity during late larval development and the post-metamorphic juvenile stage.

### Differentially expressed genes among developmental periods

**Fig. 4:**
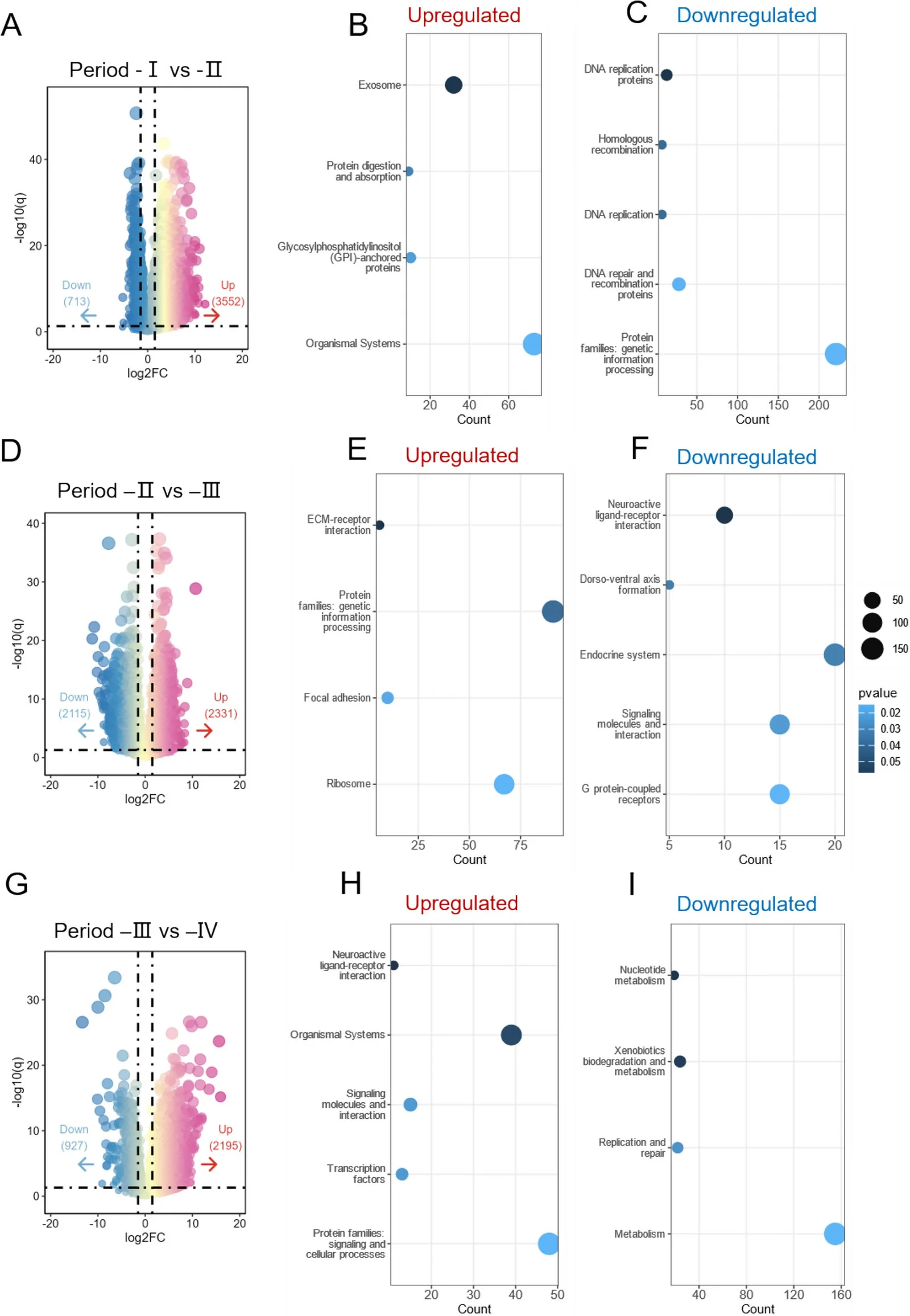
Differentially expressed genes (DEGs) and their functional enrichment analysis across the four major developmental periods. **A**, **D**, and** G** Volcano plots for DEGs between adjacent developmental periods (Period-I vs -II, Period-II vs -III, and Period-III vs -IV). Genes meeting the thresholds of abs(log2FoldChange) > 1.5 and adjusted p-value (padj) ≤ 0.01 are defined as DEGs. **B**, **E**, and** H** KEGG enrichment bubble plots for upregulated DEGs. **C**, **F**, and** I** KEGG enrichment bubble plots for downregulated DEGs. Bubble size indicates the number of enriched DEGs in each pathway (Count), and color intensity represents enrichment significance (*P*-value), with darker colors indicating higher significance

Analysis of gene expression changes during sea urchin development was conducted by comparing adjacent developmental periods, namely, Period-I to -II, Period-II to -III, and Period-III to -IV (Fig. 4A, D, and G). The Period-I to -II transition yielded 4,267 DEGs (3,552 upregulated and 715 downregulated); the Period-II to -III transition yielded 4,446 DEGs (2,331 upregulated and 2,115 downregulated); and the Period-III to -IV transition yielded 3,122 DEGs (2,195 upregulated and 927 downregulated). In the Period-I to -II transition, the number of upregulated genes was markedly greater than that of downregulated genes, whereas the differences between up- and down-regulated gene counts were less in the Period-II to -III and Period-III to -IV transitions.

### Functional enrichment analysis of differentially expressed genes

KEGG pathway enrichment analysis revealed distinct functional shifts across adjacent developmental period transitions. In the Period-I to -II transition, downregulated genes showed enrichment in genetic information processing pathways, including DNA replication, repair, and recombination, reflecting activities in genome maintenance (Fig. 4C). In contrast, upregulated genes were enriched in pathways related to organismal systems, protein digestion/absorption, GPI anchor, and exosomes, implicating early establishment of physiological functions, nutrient utilization, and cellular communication (Fig. 4B). In the Period-II to -III transition, upregulated genes were primarily enriched in the ribosome, focal adhesion and ECM-receptor interaction, implicating roles in protein synthesis and cell–matrix adhesion (Fig. 4E). Meanwhile, downregulated genes were significantly enriched in pathways such as G protein-coupled receptors, neuroactive ligand-receptor interaction, endocrine system, and dorso-ventral axis formation, which are involved in signal transduction, endocrine regulation, and developmental axis formation (Fig. 4F). In the Period-III to -IV transition, upregulated genes were mainly enriched in pathways related to signaling and cellular processes, transcription factors, signaling molecules and interaction, organismal systems, and neuroactive ligand-receptor interaction (Fig. 4H). Conversely, downregulated genes were primarily enriched in replication and repair, xenobiotics biodegradation and metabolism, and nucleotide metabolism (Fig. 4I). This pattern is consistent with enhanced signaling and transcriptional regulation alongside reduced basal metabolic and DNA maintenance activities. Moreover, the GO functional categorization of DEGs revealed patterns similar with the KEGG enrichment, and the complete GO results and detailed term lists are provided in the Supplementary Materials (Figs. S1–S6).

### Weighted gene co-expression network analysis

**Fig. 5:**
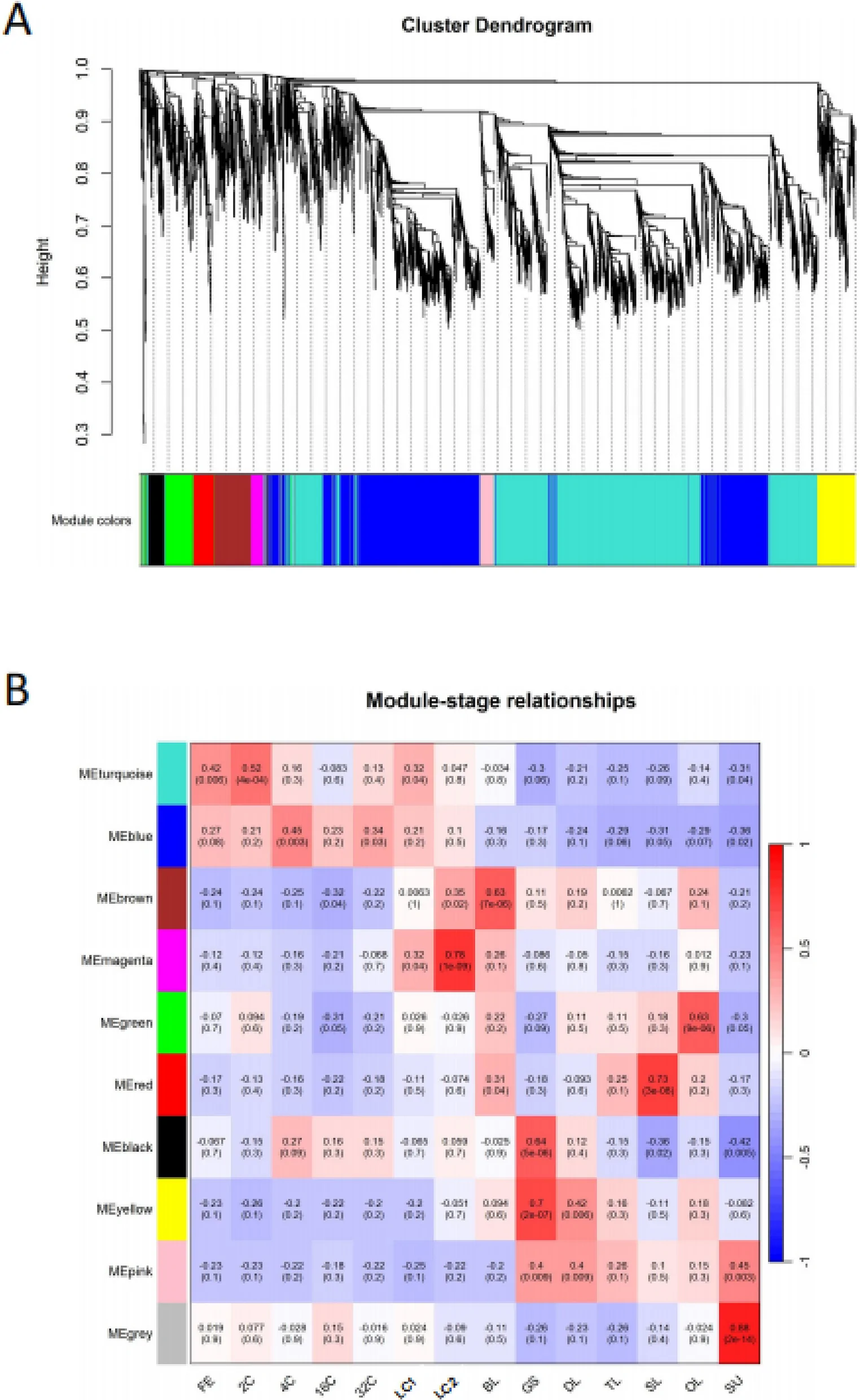
Weighted gene co-expression network analysis (WGCNA) of the *H. crassispina* developmental transcriptome. **A** Gene cluster dendrogram based on expression similarity across all 42 RNA-seq libraries, with colors denoting distinct co-expression modules. The grey module contains unassigned genes. **B** Module-trait relationship heatmap showing correlations between module eigengenes (MEs) and the 14 developmental stages. Values shown in each cell represent correlation coefficients (r) and corresponding *P*-values

To identify gene sets with coordinated expression patterns during the early development of *H. crassispina*, WGCNA was performed using all 42 samples representing 14 developmental stages. A hierarchical clustering dendrogram was generated to visualize module detection, with modules annotated in distinct colors (Fig. 5A). A total of nine co-expression modules were identified after excluding the grey module. Among these, the turquoise module contained the most genes (2,123), while the magenta module was the smallest (86). Module eigengenes were then correlated with developmental stages, and the module–trait relationships were summarized in a heatmap (Fig. 5B). The results showed that several modules were preferentially associated with specific developmental stages, including the magenta module with the late cleavage II stage (*r* = 0.78, *P <* 0.001), the brown module with the blastula stage (*r* = 0.63, *P <* 0.001), and the red module with the six-arm pluteus stage (*r* = 0.73, *P <* 0.001).

### Expression patterns of digestive enzyme genes during early development

**Fig. 6:**
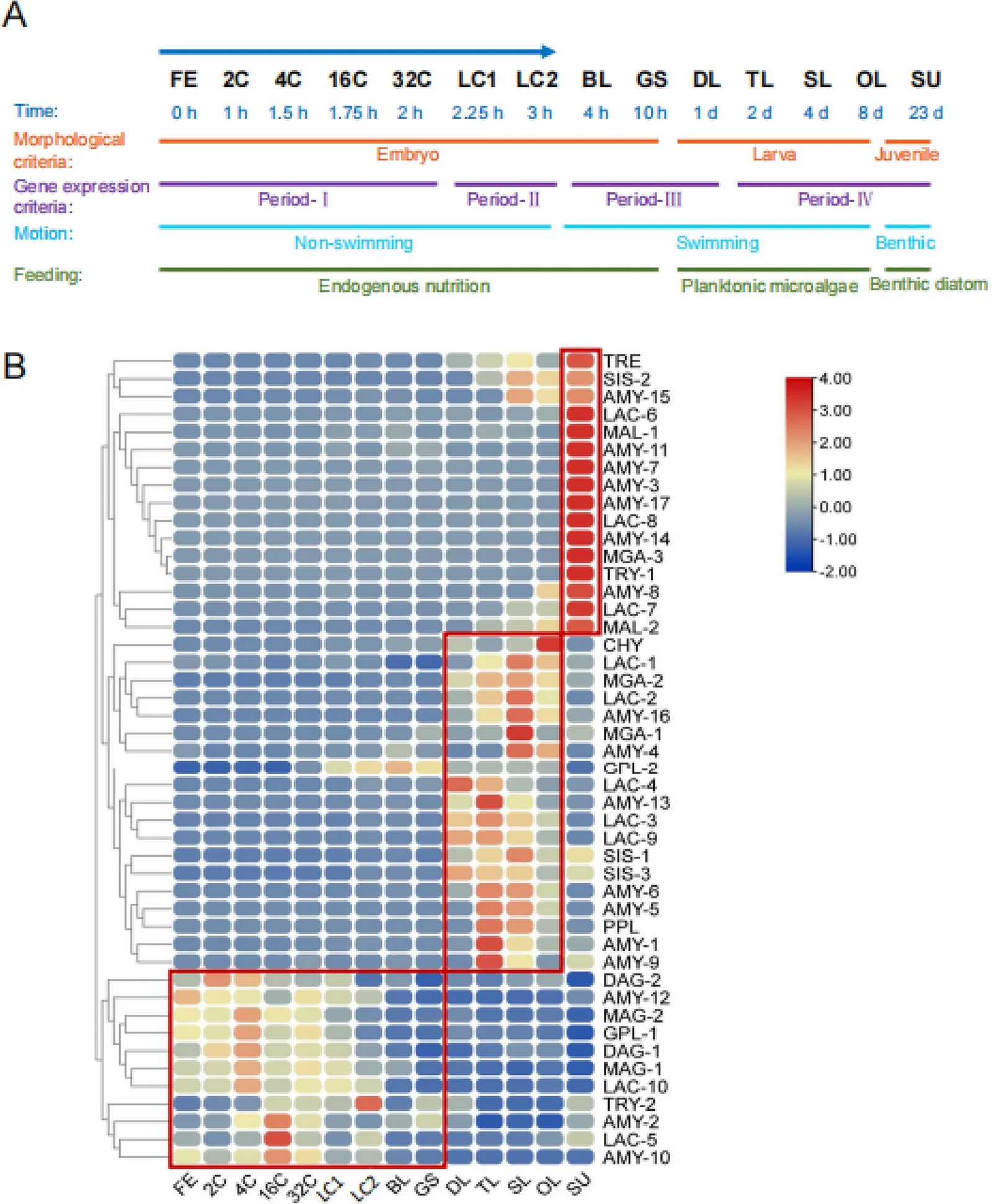
Schematic overview of development in *H. crassispina*, integrating lifestyle, feeding mode, and digestive enzyme gene expression profiles. **A** Developmental timeline from fertilized egg (FE) to juvenile (SU), showing stage abbreviations, developmental time, and stage categories by morphology (embryo, larval, juvenile), gene expression (Period-I, -II, -III, and -IV), locomotion (non-swimming, swimming, benthic), and feeding mode (endotrophic, planktonic microalgae, benthic diatoms). **B** Expression heatmap of 46 digestive enzyme genes across 14 developmental stages. Rows represent genes (labeled by enzyme abbreviation) and columns represent stages. Color intensity indicates normalized expression levels (warm for high, cool for low). Genes are hierarchically clustered, as shown by the dendrogram on the left

During development from fertilized egg to juvenile, *H. crassispina* progresses through embryonic, planktonic larval, and post-metamorphic benthic stages, accompanied by a shift in nutrient acquisition from endogenous reserves to planktonic and finally to benthic feeding (Fig. 6A). Expression profiling of the 46 digestion-related genes revealed three sequential expression modules (Fig. 6B). During embryonic stages from fertilized egg to gastrula, highly expressed genes primarily comprised enzymes for lipid digestion, including lipases and other triglyceride hydrolases, along with a small number of amylase and trypsin-like protease genes. After entering the planktonic larval stages from two-arm pluteus to pluteus stage, the high-expression profile shifted toward carbohydrate-hydrolyzing enzymes, such as α-amylase, α-glucosidase, maltase, and sucrase-isomaltase (Fig. 6B). At the stage of settled juvenile, expression of digestive enzyme shifted further, marked by a broad increase across multiple digestive enzyme categories. The most pronounced upregulation occurred in carbohydrate-hydrolyzing enzymes and proteases, accompanied by the induction of carbohydrate metabolism-related enzymes like trehalase (Fig. 6B).

### Expression patterns of genes in energy metabolism pathways during early development

**Fig. 7:**
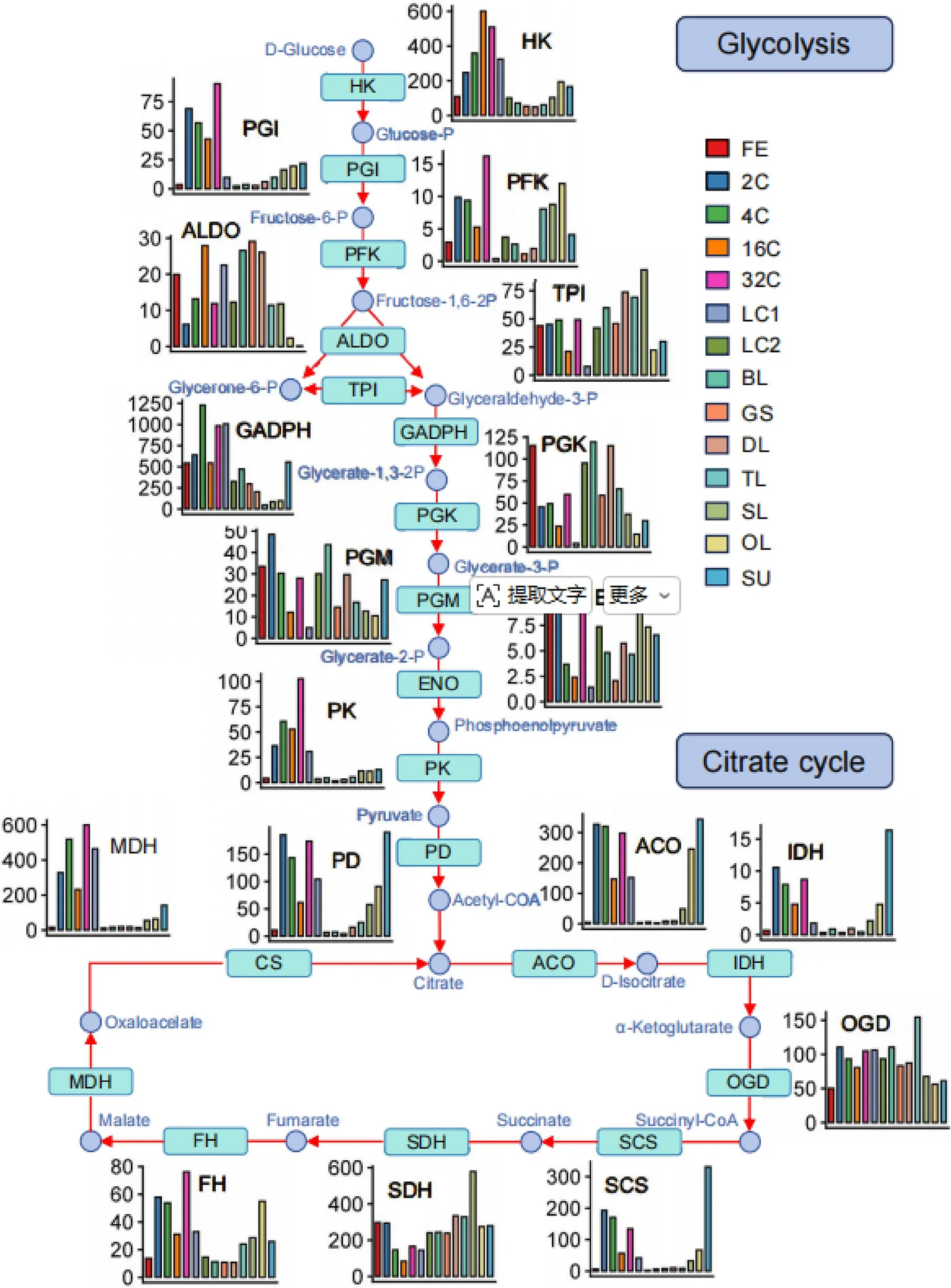
Schematic illustrating gene expression changes in key glycolytic and TCA cycle enzymes during *H. crassispina* development. Major reaction steps of glycolysis (top) and the TCA cycle (bottom) are depicted, with key enzymes labeled at their nodes. Bar charts show the expression levels of the corresponding genes across the 14 developmental stages (FE–SU). Colors represent different stages, as shown in the legend

To characterize the transcriptional regulation of energy-supply-related metabolic pathways during development, representative rate-limiting or key enzymes from major reaction steps of glycolysis and the tricarboxylic acid (TCA) cycle were selected. Their expression levels were mapped onto pathway diagrams and compared across stages from fertilized egg to juvenile (Fig. 7). The results showed that upstream glycolytic key enzymes (HK, PGI, and PFK), together with enzymes at the terminal steps of glycolysis and the mitochondrial pyruvate entry point (PK and PD), exhibited similar temporal expression patterns. Consistently, several key enzymes controlling metabolic flux in the TCA cycle (ACO, IDH, SCS, FH, and MDH) displayed coordinated changes. This indicates broadly consistent, stage-dependent expression dynamics for both glycolysis- and TCA cycle-related genes (Fig. 7).

During early development, the overall expression of multiple key enzyme genes was relatively low in the fertilized egg but increased markedly during the cleavage stages. Subsequently, expression showed a specific decline from the late cleavage II to the gastrula and remained comparatively low at the two-arm pluteus stage. From the four-arm pluteus to the juvenile stage, expression of key glycolytic and TCA-cycle enzymes increased again. Notably, genes at major flux-controlling nodes (*e.g.*, PD, ACO, IDH, and SCS) exhibited a particularly consistent upward trend. Overall, the expression of these energy-metabolism enzyme genes exhibited stage-dependent fluctuations across development, with transitions aligning with key developmental landmarks (Fig. 7).

## Discussion

By integrating transcriptomic sequencing data on gene expression dynamics with phenotypic measurements of growth and survival during *H. crassispina* embryonic and larval development, this study demonstrates that key early life-history traits do not progress linearly over time but instead undergo coordinated remodeling across several stage-specific transitions. Body length began to increase at the two-arm pluteus stage and rose significantly again from the six-arm to the eight-arm stage (Fig. 2C). In parallel, survival started to decline gradually after the two-arm pluteus stage, with the highest mortality occurring during the transition from the eight-arm pluteus to the juvenile stage through metamorphosis and attachment (Fig. 2D). Previous studies have widely reported that the shift from a planktonic lifestyle to settlement in sea urchin larvae is often accompanied by strong selective mortality and that this risk is closely associated with energy reserves, feeding and digestive capacity, and external environmental conditions [10, 21, 22]. In *H. crassispina*, mortality did not reach the peak during the cleavage, blastula, or gastrula stages, but increased after the onset of planktonic larval growth and became most pronounced during the transition from eight-arm pluteus to settled juvenile. Therefore, the late planktonic larval-to-settlement transition appears to be the main vulnerable window under the present culture conditions. The overlap between rapid body length increasing and survival declining suggests that growth, feeding, settlement preparation, and physiological remodeling occur during a period of elevated developmental risk, consistent with the major morphofunctional changes described during the perimetamorphic period of sea urchins [23].

Based on clustering analysis, the 14 developmental stages were grouped into four periods (I–IV), and KEGG enrichment of adjacent-period comparisons revealed clear stage-specific functional shifts. In the first transition (Period-I to -II), downregulated genes were enriched in genetic information processing pathways. In sea urchins and other echinoderms, the proportion of fertilized eggs that successfully complete cell division, develop into planktonic larvae, undergo metamorphosis, and ultimately settle as juveniles is typically very low, making early development a major bottleneck for population recruitment in these species [24, 25]. Meanwhile, upregulated genes were enriched in functions related to early establishment of physiological function, nutrient utilization, and cellular communication, providing a transcript foundation for the subsequent development of larval systems [26]. During the second transition (Period-II to -III), upregulated genes were enriched in protein synthesis and cell–matrix adhesion, pointing to the requirements for early organ construction [27]. In parallel, downregulated genes were enriched in developmental axis-related pathways, suggesting that axis patterning predominantly occurs in Period-II and concludes during Period-III [28].

In the final transition (Period-III to -IV), upregulated genes were enriched in pathways requiring stronger signal integration and transcriptional regulation to support behaviors, such as locomotion, environmental sensing, and food acquisition [29]. Conversely, downregulated genes were enriched in nucleotide metabolism, replication, and repair terms, indicating stage-specific adjustments in the material and energy utilization processes, accompanied by tissue remodeling associated with rapid growth [4]. Consistent with the phenotypic patterns, the planktonic larval phase represents the main period of rapid body length increase and the phase with the most pronounced decline in survival. Therefore, the transition from planktonic larva to settled juvenile within Period-IV can be regarded as a key window for optimizing feeding and rearing conditions to reduce losses.

The WGCNA results provide an organizational framework for the biological interpretation of transcriptional variation [30]. After excluding the grey module, nine co-expression modules were identified, indicating that developmental changes in *H. crassispina* embryos and larvae are coordinated at the module level rather than being dispersed across individual genes (Fig. 5A). The turquoise module contained the largest number of genes (2,123), suggesting that it may represent a broad transcriptional program shared across multiple developmental stages. In contrast, the smaller magenta module contained 86 genes and showed a strong positive correlation with the late cleavage II stage (*r* = 0.78, *P <* 0.001), suggesting a more stage-associated expression pattern. Moreover, the module-stage correlation heatmap highlighted modules preferentially associated with specific developmental stages, including the brown module with the blastula stage and the red module with the six-arm pluteus stage (Fig. 5B).

As sea urchin larvae transition from the embryonic stage reliant on endogenous nutrition to a feeding larval stage, their digestive tract gradually matures [31]. Concurrently, digestive and nutrient absorption capacities increase, and digestion-related enzyme genes often show stage-specific upregulation, thereby supporting greater energy acquisition and faster growth [32]. In *H. crassispina*, during embryonic stages from fertilized egg to gastrula, highly expressed genes primarily comprised enzymes related to lipid digestion, while a small number of amylase and trypsin genes were also maintained at relatively high levels (Fig. 6B). This pattern suggests that early development relies primarily on endogenous lipid reserves (*e.g.*, yolk) for energy, while maintaining sufficient multi-substrate hydrolytic capacity to meet the basic demands of rapid cleavage and morphogenesis. During the planktonic larval stages from two-arm to eight-arm pluteus, high expression shifted to carbohydrate-hydrolyzing enzymes, such as amylases, glycoside hydrolases, and chitinases. This shift corresponds to a feeding largely dependent on microalgae [33]. Given that microalgae and attached particles are polysaccharide-rich [34], the utilization of chitin-like substrates is often considered an adaptive supplement for exploiting particulate matter [35]. It should also be noted that this pattern was observed under a defined feeding regime using *Chaetoceros muelleri*. Therefore, the digestive enzyme-related expression changes during planktonic larval development may be influenced by the nutritional characteristics and digestibility of this algal species. The observed transcriptomic response may thus reflect, at least in part, an adaptation to the specific diet used in this study, rather than a general feature that applies equally across different algal diets or culture conditions. This developmental phase also paralleled a sustained increase in body length, suggesting that the establishment and enhancement of carbohydrate-hydrolyzing capacity may contribute to larval growth under the present feeding conditions. At the settled juvenile stage, a broad, coordinated upregulation occurred across digestive enzyme categories. Carbohydrate-hydrolyzing enzymes and proteases increased most markedly, accompanied by the induction of carbohydrate metabolism enzymes like trehalase. This expression profile is consistent with a transition toward a more complex diet, which may include benthic diatoms, biofilms, organic detritus, and macroalgal material [36, 37]. Interestingly, a similar digestive enzyme shift is reported in the sea cucumber *Holothuria leucospilota* [38]. This progression from single-category dominance to a coordinated, multi-category enhancement aligns with the benthic juvenile stage, which demands a broader substrate range and greater digestive capacity. Concurrently, survival declined steadily after the four-arm pluteus stage, reaching its lowest point at settlement. This temporal correspondence suggests that changes in digestive enzyme-related transcripts may be associated with increased larval vulnerability during metamorphosis. However, the survival decline during this transition may also reflect morphogenetic remodeling, degeneration of larval tissues, apoptosis, or other physiological stresses associated with settlement [39].

The coordinated fluctuations of key glycolytic and TCA cycle enzyme genes across development suggest stage-dependent switches in the transcriptional regulation of energy metabolism (Fig. 7). The overall elevation of the key node genes during cleavage stages indicates an increased cellular demand for energy and metabolic intermediates [40], serving dual roles in ATP production and providing biosynthetic precursors [41]. Expression of these genes subsequently declined from the late cleavage II to gastrula and remained low at the two-arm pluteus stage, implying a redistribution of metabolic flux and resources during cell-fate specification and tissue construction [42], potentially involving transient suppression of certain energy-metabolism nodes [43]. After the four-arm pluteus stage, more consistent increases in gene expression were observed at the terminal steps of glycolysis, mitochondrial pyruvate entry, and multiple TCA-cycle enzymes, suggesting further reinforcement of mitochondrial oxidative metabolism, which is potentially associated with the rising energy costs of activity, sustained growth, and tissue remodeling before and after settlement [44]. Together with the finding that digestive enzyme-related transcripts shift toward carbohydrate hydrolysis in larvae and show broad upregulation after settlement, these patterns suggest a transcriptomic association between nutrient utilization and core energy-metabolism pathways. This association may reflect increased energy demand during late larval growth and metamorphosis. However, whether these transcriptional changes correspond to changes in digestive enzyme activity, energy reserves, or metabolic flux requires further validation in future studies.

A limitation of this study is that the settled juvenile (SU) libraries were generated using a different sequencing platform from the earlier developmental stages. Consequently, potential platform-related effects cannot be completely separated from true biological differences associated with the juvenile stage. To evaluate whether the SU libraries behaved as obvious technical outliers, we examined sequencing quality metrics together with expression-level diagnostics based on stable expressed genes. These analyses did not identify systematic abnormalities in sequencing quality, expression shift, within-stage variability, or replicate consistency. Nevertheless, these diagnostics cannot eliminate the platform × stage confounding. Therefore, transcriptomic patterns that are primarily driven by the SU stage, particularly those involving digestive enzyme genes, energy-metabolism genes, and functional enrichment analyses, should be interpreted with appropriate caution.

## Conclusion

This study presents an integrated analysis of the gene expression dynamics, together with growth and survival of *H. crassispina* from fertilized egg to juvenile stages, revealing clear stage-specific transitions in its early life history. Body length began to increase at the two-arm pluteus stage and showed a further marked rise from the six-arm to eight-arm stages. In parallel, survival declined continuously after the four-arm pluteus stage, with the highest mortality occurring during the transition from the eight-arm pluteus to the juvenile stage. KEGG enrichment results indicate that early development is dominated by genetic information maintenance processes, middle development is characterized by enhanced protein synthesis and cell–matrix adhesion, whereas late development shows strengthened signal transduction and transcriptional regulation, highlighting the importance of optimizing stage-specific feeding and rearing conditions management. The expression of digestive enzyme genes shifted sequentially, as lipid-digestion enzymes dominated early stages; carbohydrate-hydrolyzing enzymes became predominant during the planktonic larval phase; and multiple enzyme classes were coordinately enhanced after settlement, along with enzymes for carbohydrate metabolism, reflecting an expansion of nutrient sources and substrate spectrum during development. Key node genes in glycolysis and the TCA cycle exhibited stage-dependent fluctuations and increased overall during late larval stages, suggesting a close link between energy-metabolism regulation and processes of growth and metamorphosis. Overall, this study describes the dynamic regulation of nutrient utilization and energy metabolism in early development of *H. crassispina*, providing a basis for improvement of hatchery management and stage-appropriate feeding.

## Supplementary Information

Supplementary Material 1: Supplementary_Figures_GO_enrichment_FigS1-S6.

Supplementary Material 2: Supplementary_Fig_S7_soft_thresholding_power_selection.

Supplementary Material 3: Supplementary_Fig_S8_size_measurement_schematic.

Supplementary Material 4: Supplementary_Table_S1.

Supplementary Material 5: Supplementary_Table_S2_PCA_PC1_PC2_factor_loadings.

Supplementary Material 6: Supplementary_Table_S2_PCA_PC1_PC2_factor_loadings.

Supplementary Material 7: Supplementary_Table_S4_SU_technical_outlier_diagnostic_report.

## Data Availability

The raw RNA-seq reads generated in this study have been deposited in the NCBI Sequence Read Archive (SRA) under BioProject accession PRJNA1413484. In the SRA metadata, samples originally labeled as MC and MO correspond to the revised stage names late cleavage I (LC1) and late cleavage II (LC2), respectively.
