# Supplementary material for: Transcriptomic sequencing reveals dynamic shifts in nutrient utilization and energy metabolism during embryonic and larval development of the purple sea urchin Heliocidaris crassispina
Source: BMC Genomics. 2026 Jul 30;27:887. doi: 10.1186/s12864-026-13190-z (PMC13711962; doi:10.1186/s12864-026-13190-z)
Supplement: Supplementary file 1 [file 12864_2026_13190_MOESM1_ESM.pdf]

Fig.1

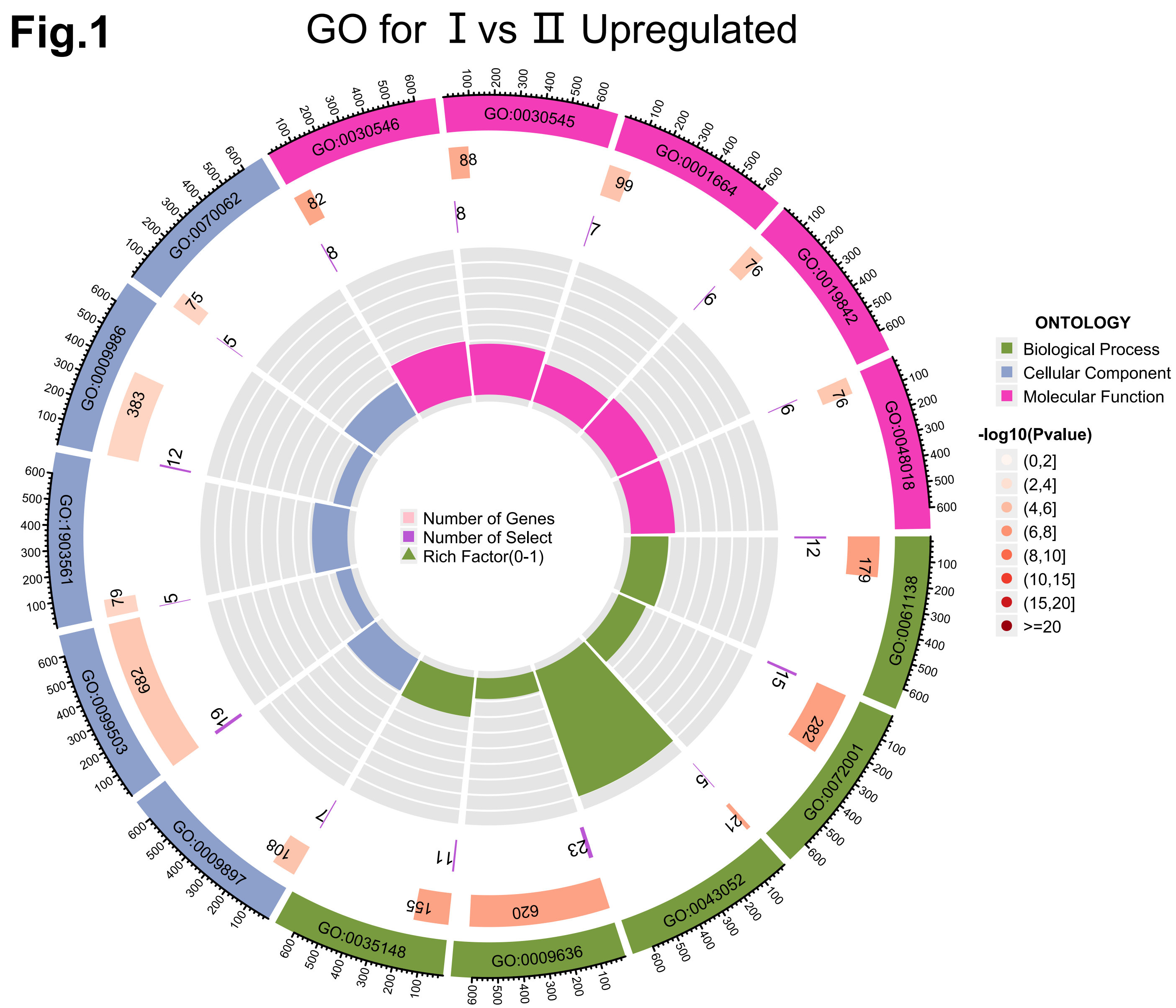

ONTOLOGY	ID	Description.x
MF	GO:0030546	signaling receptor activator activity
MF	GO:0030545	signaling receptor regulator activity
MF	GO:0001664	G protein-coupled receptor binding
MF	GO:0019842	vitamin binding
MF	GO:0048018	receptor ligand activity
CC	GO:0009897	external side of plasma membrane
CC	GO:0099503	secretory vesicle
CC	GO:1903561	extracellular vesicle
CC	GO:0009986	cell surface
CC	GO:0070062	extracellular exosome
BP	GO:0061138	morphogenesis of a branching epithelium
BP	GO:0072001	renal system development
BP	GO:0043052	thermotaxis
BP	GO:0009636	response to toxic substance
BP	GO:0035148	tube formation

Fig.2

GO for Ⅰ vs Ⅱ Downregulated

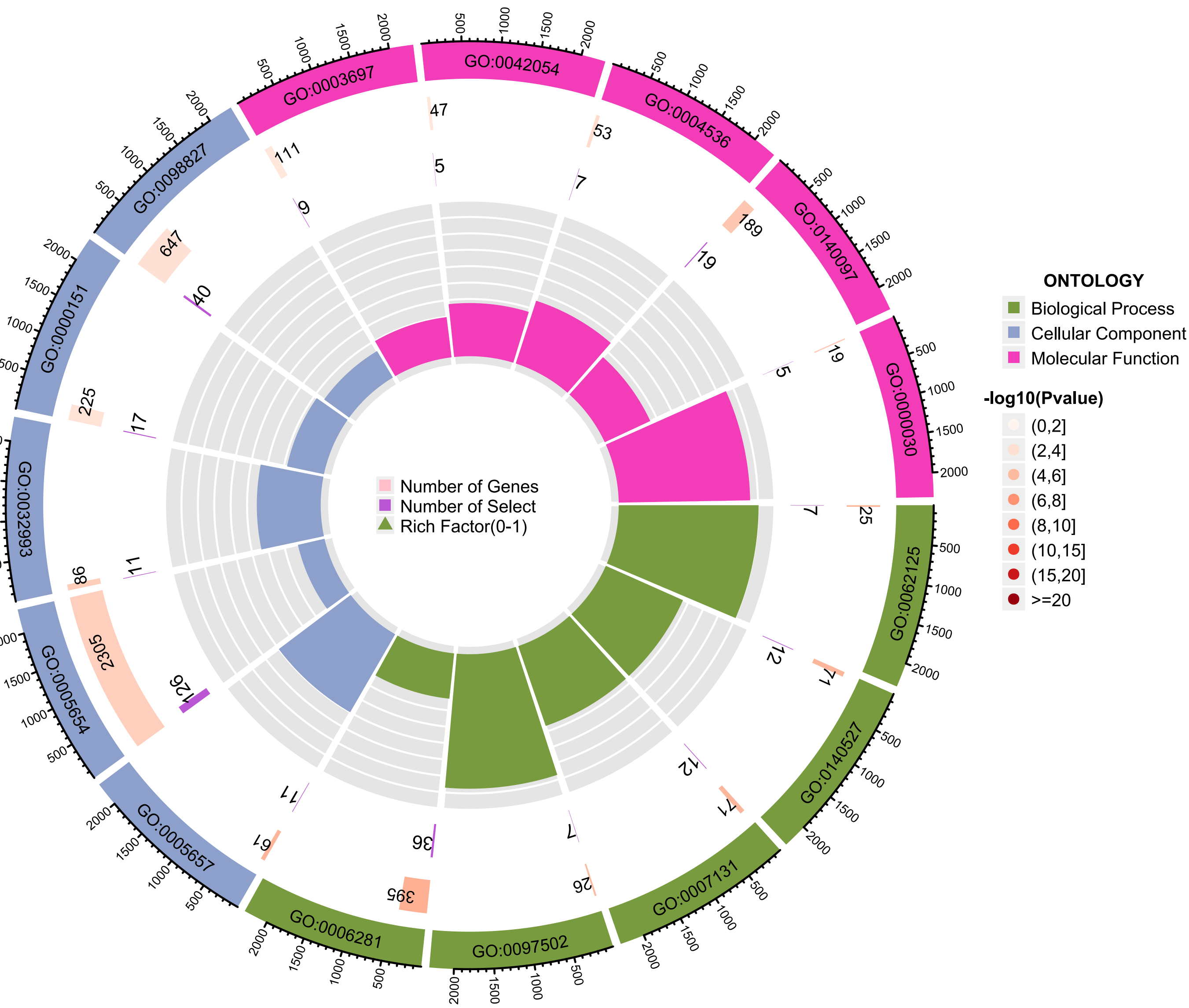

ONTOLOGY	ID	Description.x
MF	GO:0003697	single-stranded DNA binding
MF	GO:0042054	histone methyltransferase activity
MF	GO:0004536	DNA nuclease activity
MF	GO:0140097	catalytic activity, acting on DNA
MF	GO:0000030	mannosyltransferase activity
CC	GO:0005657	replication fork
CC	GO:0005654	nucleoplasm
CC	GO:0032993	protein-DNA complex
CC	GO:0000151	ubiquitin ligase complex
CC	GO:0098827	endoplasmic reticulum subcompartment
BP	GO:0062125	regulation of mitochondrial gene expression
BP	GO:0140527	reciprocal homologous recombination
BP	GO:0007131	reciprocal meiotic recombination
BP	GO:0097502	obsolete mannosylation
BP	GO:0006281	DNA repair

Fig.3

GO for Ⅱ vs Ⅲ Upregulated

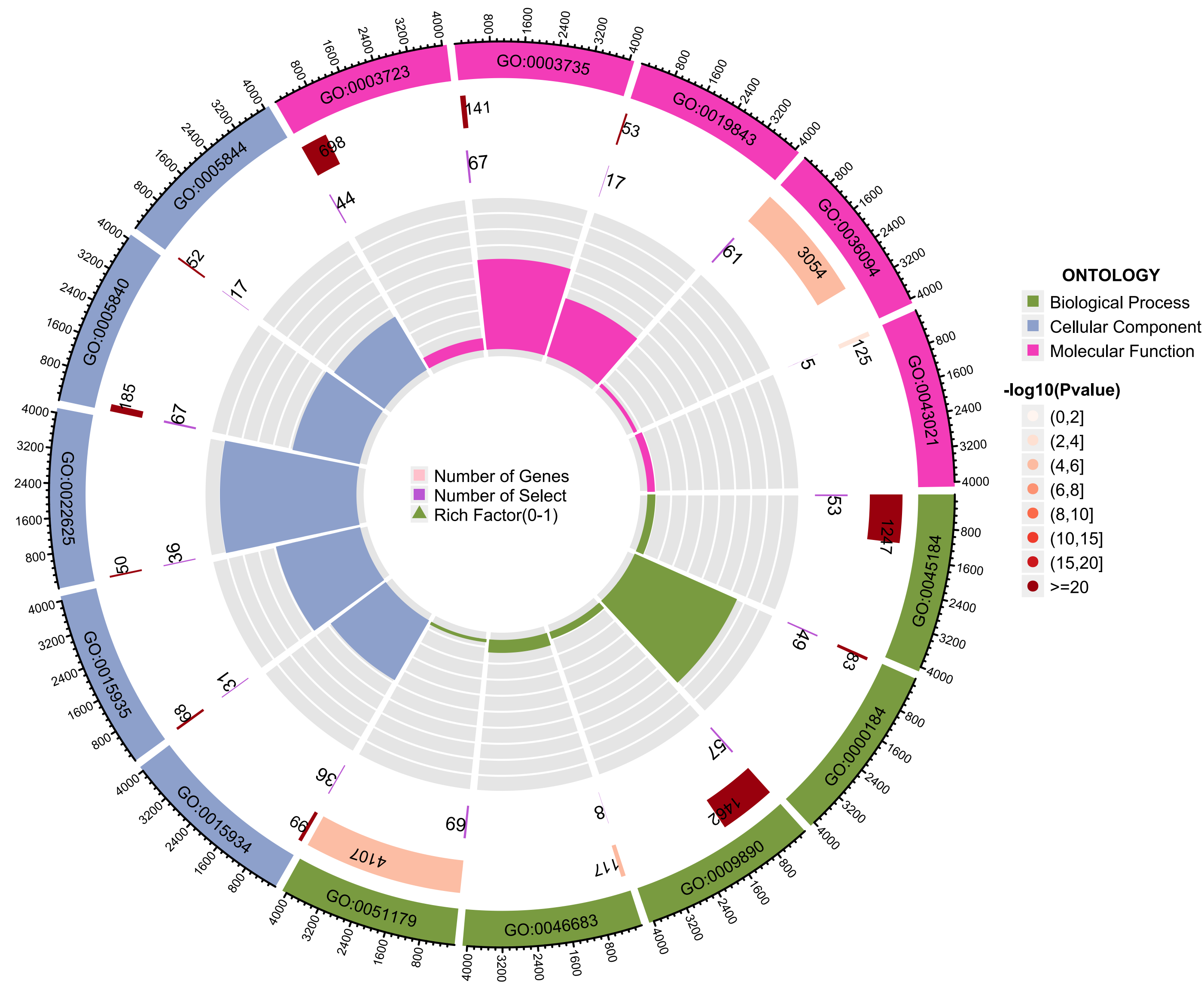

ONTOLOGY	ID	Description.x
MF	GO:0003723	RNA binding
MF	GO:0003735	structural constituent of ribosome
MF	GO:0019843	rRNA binding
MF	GO:0036094	small molecule binding
MF	GO:0043021	ribonucleoprotein complex binding
CC	GO:0015934	large ribosomal subunit
CC	GO:0015935	small ribosomal subunit
CC	GO:0022625	cytosolic large ribosomal subunit
CC	GO:0005840	ribosome
CC	GO:0005844	obsolete polysome
BP	GO:0045184	establishment of protein localization
BP	GO:0000184	nuclear-transcribed mRNA catabolic process, nonsense-mediated decay
BP	GO:0009890	negative regulation of biosynthetic process
BP	GO:0046683	response to organophosphorus
BP	GO:0051179	localization

Fig.4

GO for Ⅱ vs Ⅲ Downregulated

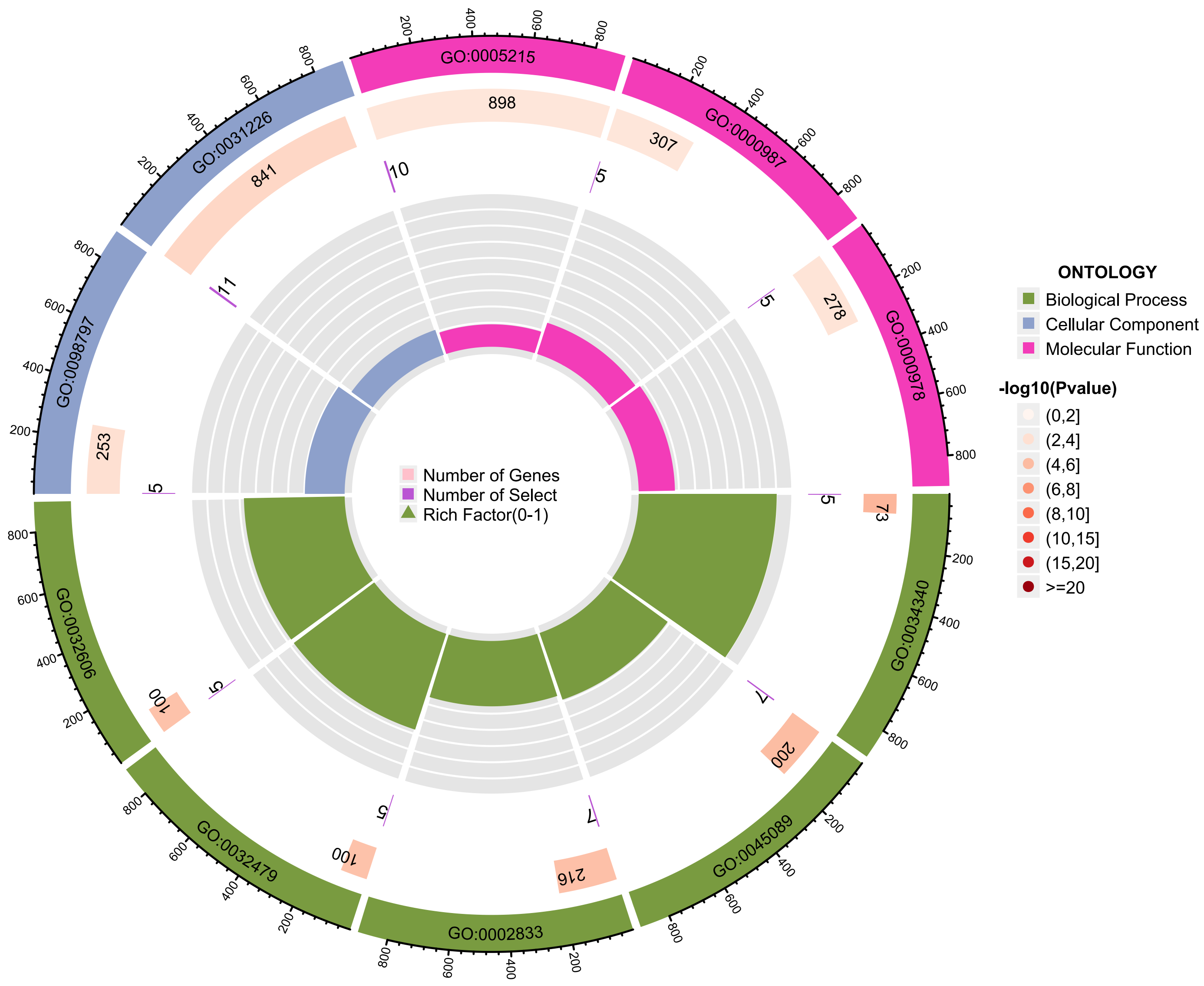

ONTOLOGY	ID	Description.x
MF	GO:0000978	RNA polymerase II cis-regulatory region sequence-specific DNA binding
MF	GO:0000987	cis-regulatory region sequence-specific DNA binding
MF	GO:0005215	transporter activity
CC	GO:0031226	obsolete intrinsic component of plasma membrane
CC	GO:0098797	plasma membrane protein complex
BP	GO:0034340	response to type I interferon
BP	GO:0045089	positive regulation of innate immune response
BP	GO:0002833	positive regulation of response to biotic stimulus
BP	GO:0032479	regulation of type I interferon production
BP	GO:0032606	type I interferon production

Fig.5

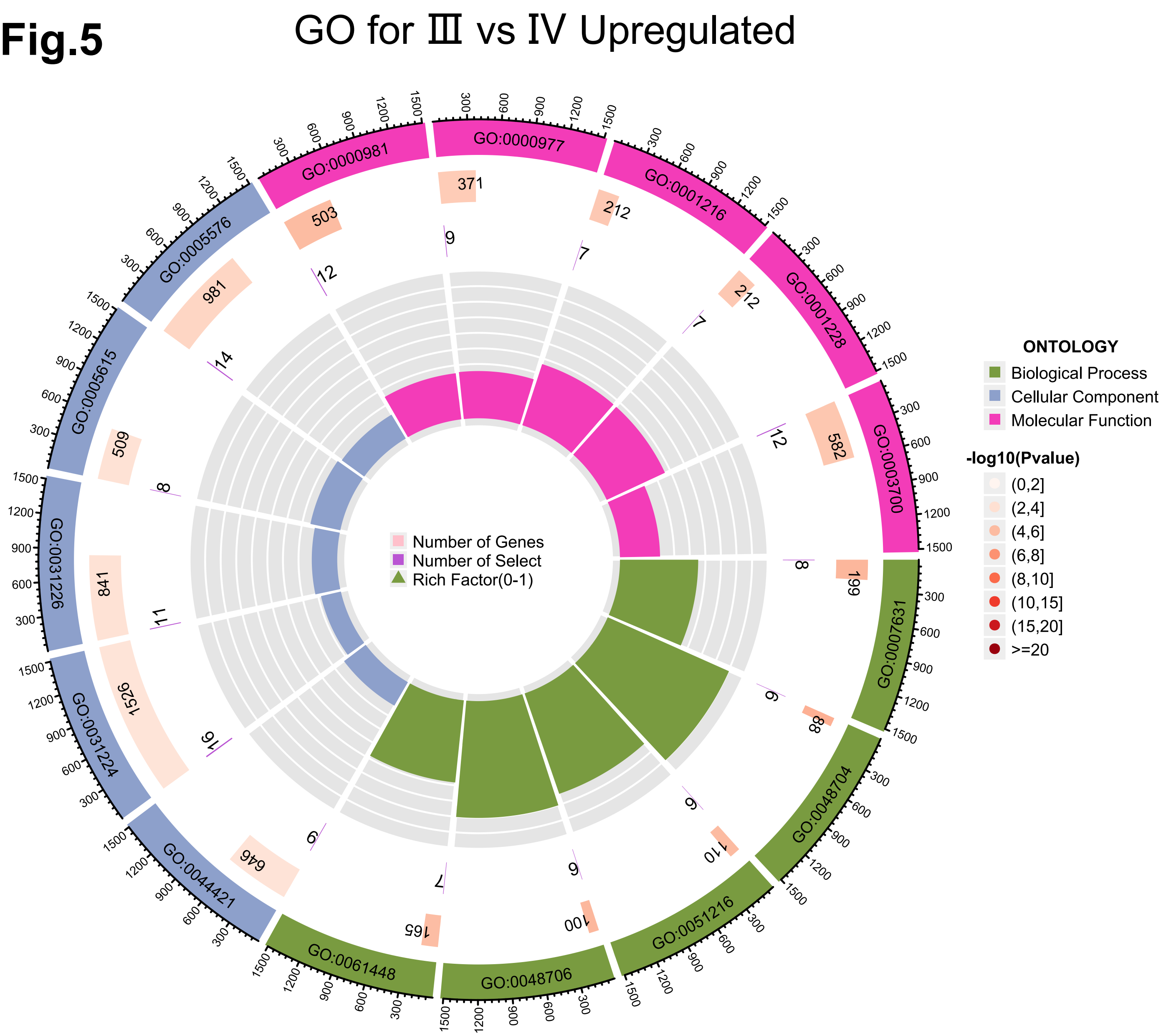

ONTOLOGY	ID	Description.x
MF	GO:0000981	DNA-binding transcription factor activity, RNA polymerase II-specific
MF	GO:0000977	RNA polymerase II transcription regulatory region sequence-specific DNA binding
MF	GO:0001216	DNA-binding transcription activator activity
MF	GO:0001228	DNA-binding transcription activator activity, RNA polymerase II-specific
MF	GO:0003700	DNA-binding transcription factor activity
CC	GO:0005576	extracellular region
CC	GO:0005615	extracellular space
CC	GO:0031226	obsolete intrinsic component of plasma membrane
CC	GO:0044421	obsolete extracellular region part
CC	GO:0031224	obsolete intrinsic component of membrane
BP	GO:0007631	feeding behavior
BP	GO:0048704	embryonic skeletal system morphogenesis
BP	GO:0051216	cartilage development
BP	GO:0048706	embryonic skeletal system development
BP	GO:0061448	connective tissue development

Fig.6

GO for Ⅲ vs Ⅳ Downregulated

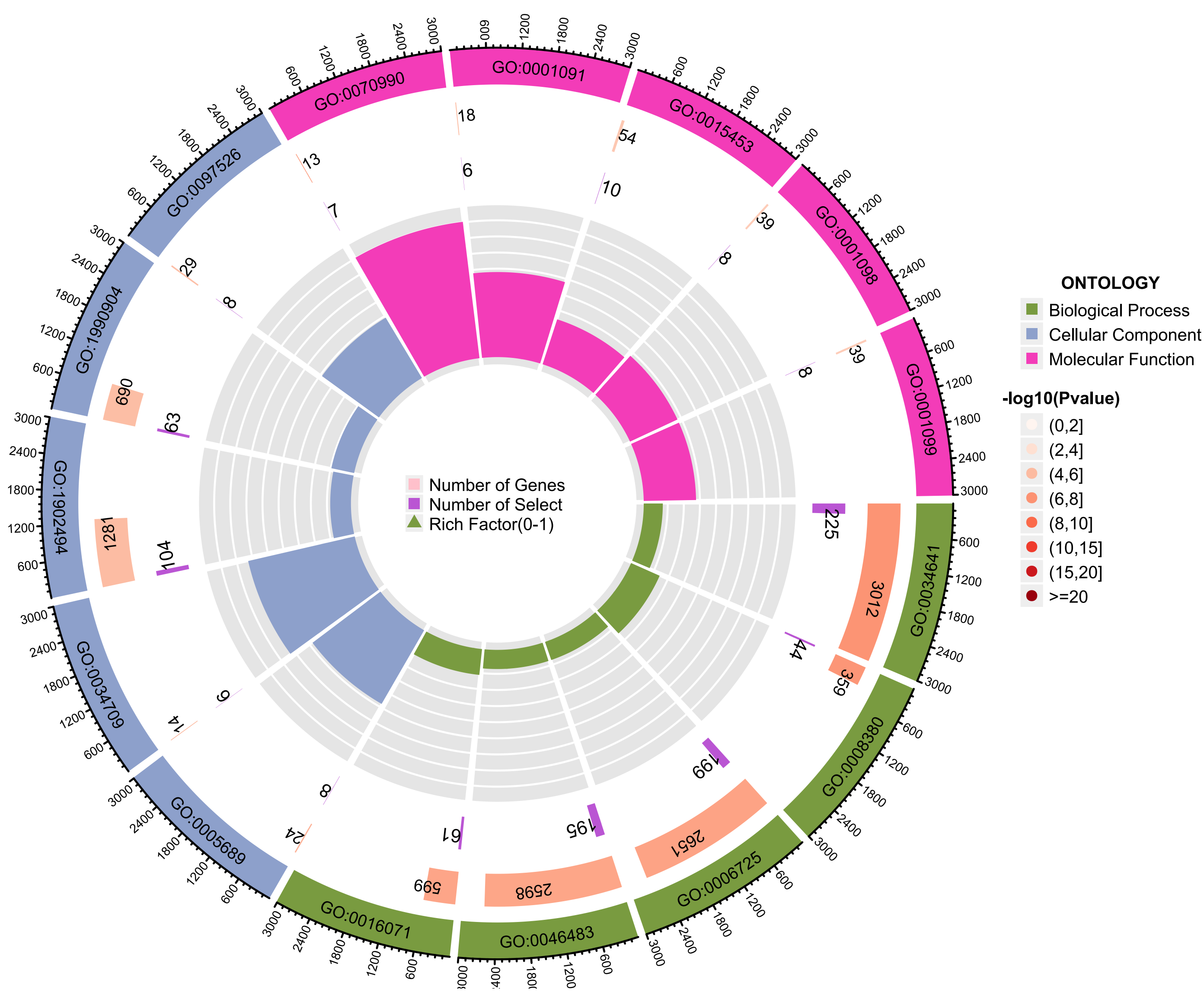

ONTOLOGY	ID	Description.x
MF	GO:0070990	snRNP binding
MF	GO:0001091	RNA polymerase II general transcription initiation factor binding
MF	GO:0015453	oxidoreduction-driven active transmembrane transporter activity
MF	GO:0001098	basal transcription machinery binding
MF	GO:0001099	basal RNA polymerase II transcription machinery binding
CC	GO:0005689	U12-type spliceosomal complex
CC	GO:0034709	methylosome
CC	GO:1902494	catalytic complex
CC	GO:1990904	ribonucleoprotein complex
CC	GO:0097526	spliceosomal tri-snRNP complex
BP	GO:0034641	obsolete cellular nitrogen compound metabolic process
BP	GO:0008380	RNA splicing
BP	GO:0006725	obsolete cellular aromatic compound metabolic process
BP	GO:0046483	obsolete heterocycle metabolic process
