# Supplementary material for: Transcriptomic sequencing reveals dynamic shifts in nutrient utilization and energy metabolism during embryonic and larval development of the purple sea urchin Heliocidaris crassispina
Source: BMC Genomics. 2026 Jul 30;27:887. doi: 10.1186/s12864-026-13190-z (PMC13711962; doi:10.1186/s12864-026-13190-z)
Supplement: Supplementary file 2 [file 12864_2026_13190_MOESM2_ESM.pdf]

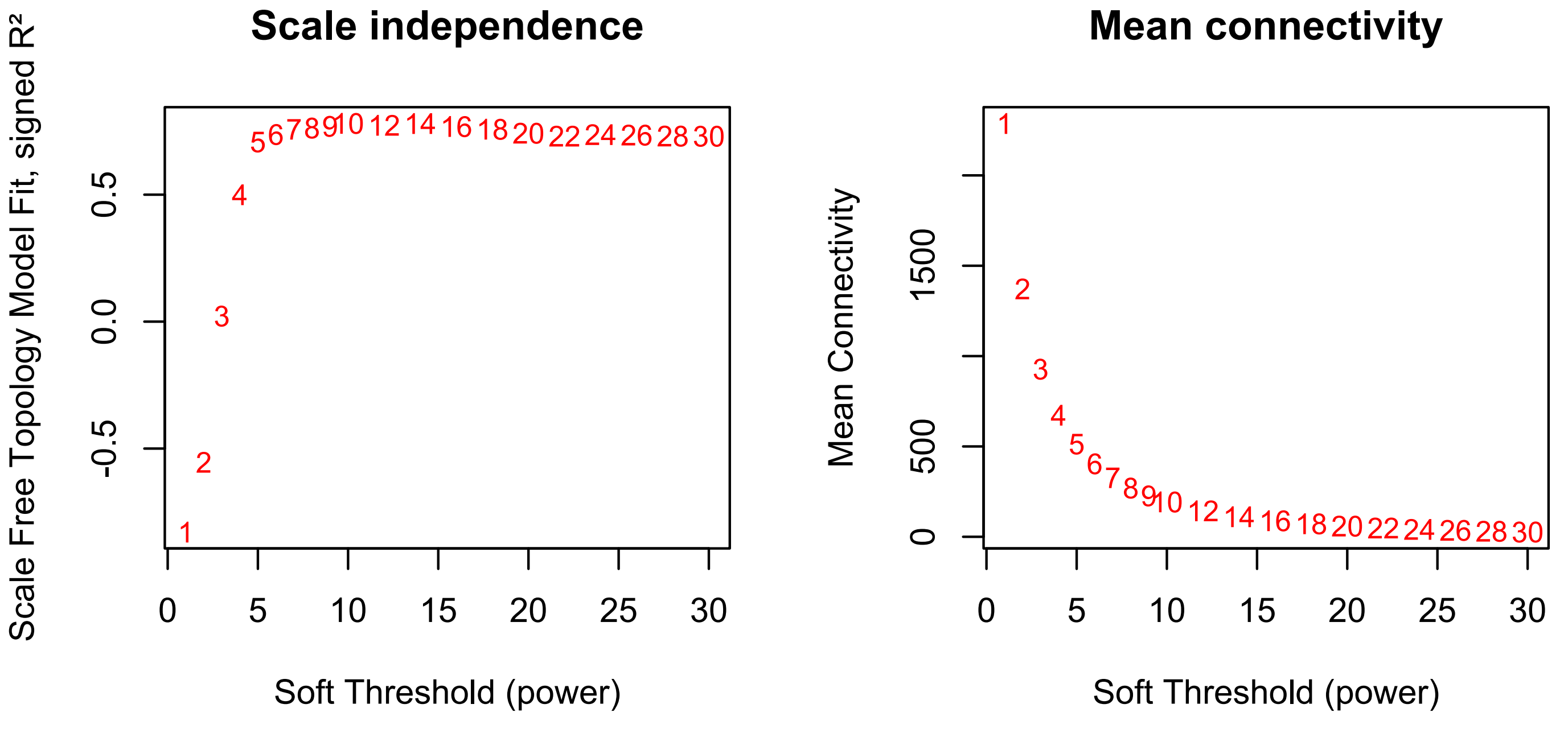

Scale independence
Scale Free Topology Model Fit, signed R²
-0.5
0.0
0.5
Soft Threshold (power)
0
5
10
15
20
25
30
Mean connectivity
Mean Connectivity
0
500
1500
Soft Threshold (power)
