# Supplementary material for: Transcriptomic sequencing reveals dynamic shifts in nutrient utilization and energy metabolism during embryonic and larval development of the purple sea urchin Heliocidaris crassispina
Source: BMC Genomics. 2026 Jul 30;27:887. doi: 10.1186/s12864-026-13190-z (PMC13711962; doi:10.1186/s12864-026-13190-z)
Supplement: Supplementary file 3 [file 12864_2026_13190_MOESM3_ESM.pdf]

Fig.7

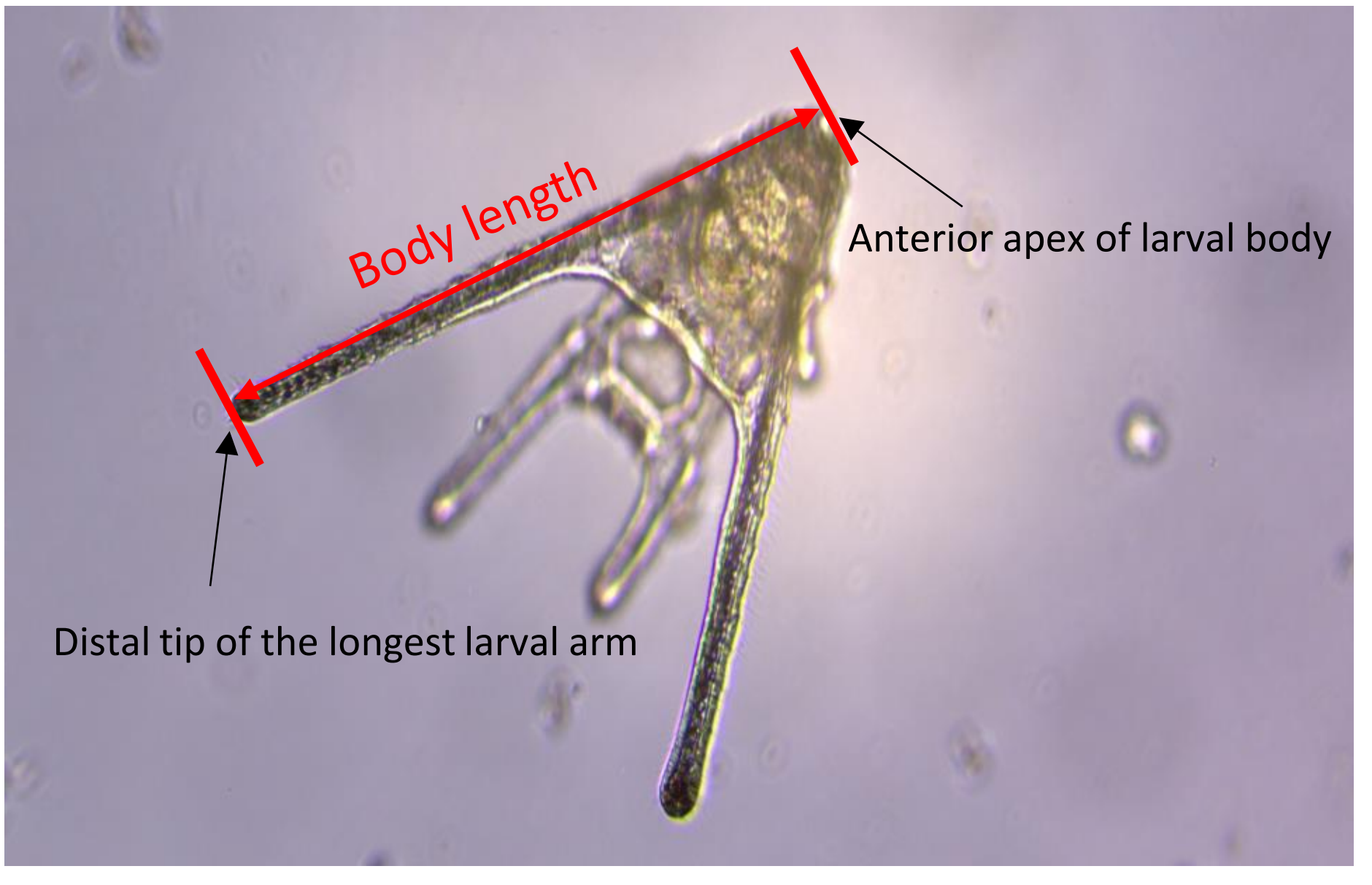
Body length
Anterior apex of larval body
Distal tip of the longest larval arm
